# *Mycobacterium genavense* Infections in a Tertiary Hospital and Reviewed Cases in Non-HIV Patients

**DOI:** 10.1155/2014/371370

**Published:** 2014-02-19

**Authors:** M. Santos, A. Gil-Brusola, A. Escandell, M. Blanes, M. Gobernado

**Affiliations:** ^1^Arevalo Baca Street 3, 46010 Valencia, Spain; ^2^Department of Microbiology, University Hospital La Fe, Avenida Campanar 21, 46009 Valencia, Spain; ^3^Infectious Diseases Unit, University Hospital La Fe, 46009 Valencia, Spain

## Abstract

*Mycobacterium genavense* is a relatively new species of nontuberculous mycobacterium reported to cause disseminated infections in patients with AIDS and later on in non-HIV immunosuppressed patients. We describe clinical and laboratory features and response to therapy in 7 patients, three of them with HIV infection and four non-HIV—three organ transplant recipients and one with hyper-IgE syndrome—in Valencia, Spain, in a ten years period. We then summarize the published cases of *M. avium complex* infection, with invasion of peripheral blood, liver, spleen, bone marrow, lymph nodes, and lungs. In clinical samples a large number of acid-fast bacilli were observed. *M. genavense* grew only from liquid media and after a prolonged incubation period. Its identification was accomplished through molecular methods. Patients were treated with prolonged combinations of antimicrobial agents. There was clinical favourable outcome in 4 patients.

## 1. Introduction

*Mycobacterium genavense* is a nontuberculous mycobacterium (NTM), first described in 1990 [1], proposed as a new species in 1992 [2, 3], and characterized in 1993 [4]. It is a slowly growing fastidious mycobacterium that has been found in tap water [5], animals (birds, rabbits, cats, ferrets, and rabbits) [6–9], and intestinal tract of healthy humans [10]. No human to human transmission has been demonstrated. It can cause infection in birds—it is the most frequently isolated mycobacterium in parrots and parakeets [11, 12]—and humans [13–16]. In the latter, symptoms vary from nonspecific in otherwise healthy patients to disseminated symptomatology in immunosuppressed ones. These are similar to those observed in *Mycobacterium avium complex *(MAC) infection and can include fever, abdominal pain, diarrhea, weight loss, lymphadenitis, hepatosplenomegaly, and progressive anemia, being the bowel the most affected organ [17]. Other less frequently involved organs are the lungs [18], central nervous system [19], skin and soft tissues [20, 21], and genital tract [22]. Its tendency to colonize the small intestine suggests that the digestive tract could act as a reservoir and that transmission could be oral or intestinal [10].

Most reports of *M. genavense* infection are from the pre-HAART era, in AIDS patients from Europe [14, 16, 23, 24], America [3, 13, 25], Asia [26], and Australia [27, 28]. Since 1997, few cases in non-HIV patients have been reported, mainly in immunosuppressed patients, including solid organ transplant recipients [23, 29], and patients with lymphoproliferative malignancies [30] or other immunosuppressive therapy [31, 32].

*M. genavense* is a fastidious NTM that needs liquid media, acid pH, higher than usual temperature (45°C), mycobactin J as supplement, and at least 3 months of incubation to grow [3, 15, 17, 33]. Molecular methods are needed for its definitive identification to a species level [2, 17, 24, 34]. Nevertheless, these are also difficult since this species is related, from a phylogenetic point of view, to *M. simiae* and *M. malmoense *[35, 36]. Susceptibility testing of *M. genavense* is also arduous, not only due to its problematical isolation, but also due to the large incubation period required for its growth [4, 15, 17]. Several studies inform that it is resistant to isoniazid with variable results with ethambutol and susceptible to streptomycin, rifampicin, clarithromycin, amikacin, fluoroquinolones, and pyrazinamide [17, 37]. Even though its optimal treatment is not set, combinations of at least three active drugs are used, similarly to other NTM infections.

We hereon present a retrospective analysis of the clinical and microbiological aspects of the cases of *M. genavense* infection diagnosed in a tertiary hospital in Valencia, Spain, between 2003 and 2013, and also review the published cases from non-HIV patients in order to describe the clinical profile of this type of infection.

## 2. Materials and Methods

Retrospective search through the database of the Microbiology Department of the University Hospital La Fe in Valencia, Spain, of all isolates identified *M. genavense* between 2003 and 2013. This hospital has around 1000 beds with a catchment area of 360,000 inhabitants including 20 primary care centres. Data recovered included type of samples, culture and identification methods, and susceptibility results. The medical records of all patients with *M. genavense* isolation in any clinical sample were reviewed. Data collected included age, sex, underlying diseases, immune status with CD4 lymphocyte count, radiological, radioscopic and histological findings, treatment received, and outcome.

Microbiologic processing followed the standard protocol of the laboratory. Samples from nonsterile sites were decontaminated using the N-acetyl-L-cysteine-sodium hydroxide treatment. Staining with Ziehl-Neelsen method was always done. The samples were cultured in two Löwenstein-Jensen (LJ) solid media and liquid media: either BACTEC 12B Mycobacteria Medium which contains 4 mL of Middlebrook 7H12 to be periodically measured in the BACTEC 460TB radiometric instrument until 2008 or the BBL Mycobacteria Growth Indicator Tube (MGIT) with 7 mL modified Middlebrook 7H9 with the BD MGIT 960 automated fluorometric system, from that year onwards. Blood samples and bone marrow were directly inoculated in 13A radiometric medium until the year 2008 and later into BACTEC MYCO/F Lytic medium designed for use with the BACTEC 9050 automated system. Incubation was routinely done at 37°C for at least 6 weeks, extended to 3 or even 4 months when this mycobacterium was suspected. Processing of positive cultures included Ziehl-Neelsen stain, subculturing to a LJ with and without pyruvate, chocolate, Sabouraud dextrose, and blood agar plates incubated at both 37°C and 42°C. Identification was partially accomplished through biochemical methods but was then definitively made through DNA probes (GenoType HAIN Lifescience) or 16S rRNA gene sequencing. Antibiotic susceptibility testing in liquid media (BACTEC 12B Mycobacteria Medium or BD MGIT) included isoniazid, rifampicin, streptomycin, ethambutol, and pyrazinamide drugs.

To assess lung infection caused by *M. genavense* we used the American Thoracic Society (ATS) 2007 criteria [37], and for the intestinal infection we considered intestinal isolates in several successive samples in patients with prolonged symptoms. Disseminated infection was considered as the presence of signs or symptoms involving two or more organs or systems and/or isolation of *M. genavense* in blood, bone marrow, or other organs.

For the review of non-HIV cases published in literature, information from Medline databases (PubMed and Ovid) was used.

## 3. Results

In the period of study, 7 cases of *M. genavense* infection were identified, three HIV positive and four non-HIV.  Table 1 shows demographic data of the patients, together with information concerning their underlying diseases, main symptoms, radiological findings, antibiotic treatment, and final outcome. Pathologic findings from biopsies (jejunal, duodenal lymph nodes, lung, and spleen) showed inflammatory changes, granulomas, and a large amount of intracytoplasmatic AFB, compatible with atypical mycobacteriosis. The three AIDS patients were receiving antiretroviral treatment and cotrimoxazole prophylaxis for *Pneumocystis jirovecii,* the three transplant recipients were on immunosuppressive drugs, and the child had no immunosuppressive therapy. The interval between start of immunosuppression and the *M. genavense* diagnosis was of 4 years in case 4, 1 year in case 5, and 6 years in case 6. In the rest of patients, initiation of immunosuppression is difficult to determine since they were attended at our hospital in a late stage of HIV infection. All patients had disseminated infection, five of them with symptoms and/or radiological images compatible with pulmonary infection, three (cases 2, 6 and 7) according to ATS criteria [37] and isolation of *M. genavense*.

Table 2 collects year of isolation, microbiological aspects such as type and number of samples cultured, whether AFB were observed in the initial stain, culture medium where it grew, in which samples was the mycobacterium isolated or was DNA detected, and *in vitro* drug susceptibility results. Culturing in liquid broth was always successful, being not the case of the solid LJ medium, in which *M. genavense* did not grow neither from the original samples nor from the subcultures. The identification at species level, in order to exclude *M. tuberculosis* and other NTM, was always done by conventional and molecular methods. All strains of *M. genavense* were urease producers.

Table 3 shows the cumulative cases of *M. genavense* infection in non-HIV patients published in the scientific literature from 1997 to present, including four of this study.

## 4. Discussion

All documented infections by *M. genavense *have been in immunocompromised hosts, mainly AIDS patients with CD4 count below 50/*μ*L in the pre-HAART era, except for one reported case of lymphadenitis in a healthy human [44]. The incidence of *M. genavense* infection in the Swiss HIV cohort study was found to be 12.8% of all NTM cases, making it the second most frequent NTM, after MAC [14]. However, data concerning the frequency of infection caused by this mycobacterium is few and disperse, probably due to its difficult clinical suspicion and microbiological isolation, so the disease might be underdiagnosed. In our series, the 7 patients had a diminished immune system: 3 HIV positive, 3 transplant recipients, and one child with hyper-IgE syndrome. In the different studies more cases of infection were found in adults [14, 16, 23], being less frequent in children [44–46] and with no discrimination concerning sex.

The majority of reported cases are disseminated, so clinicians should suspect this disease when a patient presents symptoms similar to those of disseminated MAC infection. *M. genavense* often affects the bowel and has less frequently been related to pleuropulmonary involvement [18, 20, 29, 47]. Other less commonly reported localizations are cutaneous, cerebral as a solitary lesion, and genital tract [19, 20, 22]. It sometimes appears associated with other opportunistic infections, making its diagnosis even more difficult. In our series, all patients had disseminated infections with intestinal tract invasion. Pulmonary infection was proofed with isolation of the mycobacterium in three cases, even though five patients had fever and productive cough and four had altered chest X ray.

Clinical presentation in affected patients usually includes fever, diarrhea, weight loss, abdominal pain, swelling of lymph nodes, hepatosplenomegaly, progressive anemia, low CD4 lymphocyte count, and sometimes hyperammonemia [38]. All these signs and symptoms are difficult to differentiate from disseminated MAC infection, except for the abdominal pain, present in the seven cases in our series, which is more frequent in *M. genavense* infection [17].

Pathologic findings from spontaneous samples and biopsies show inflammatory changes, granulomas, and a large amount of intracytoplasmatic AFB, all indistinguishable from MAC infection [15, 23, 48], in accordance with our results.

Microbiological findings are also similar to those of MAC infection. Samples like faeces and biopsies show a large amount of AFB. These are small, thin coccobacilli and appear disperse in direct staining from samples and accumulated when cultured. In our series, we observed these same facts in all stools and biopsies (retroperitoneal, duodenal, lung, and spleen).

Culture in liquid broth is preferred, BACTEC 13A medium as a good classic option [3]. Subculture in solid media is accomplished after adjusting pH, supplementing Mycobactin J, better on Middlebrook 7H11 agar than in LJ, in microaerophilic conditions, and incubation temperature of 37–45°C [3, 4, 49]. It is important to suspect *M. genavense* and incubate the samples for a longer period of time, even up to 4 months if AFB were observed in the direct staining of the sample. Our strains grew only in liquid medium after a 3-4-month period of incubation, since we do not use supplemented solid media. Neither an enriched CO_2_ atmosphere nor a higher temperature facilitated the growth of the mycobacterium. It is important to consider a prolonged incubation period, especially when using automated machines with closed protocols. Blood and bone marrow, biopsies, and stools were samples that showed good microbiological results—the first ones probably due to their direct inoculation in the liquid media without previous processing and the stools because of their abundant AFB load.

During the period of study, there were some samples with acid-fast coccobacilli similar to *M. genavense*, which neither grew nor were identified. These could have been *M. genavense* but also other subspecies of MAC that have similar clinical presentation and are also difficult to grow and mycobactin J dependent [3].

Biochemical identification to species level is problematic, being urease slowly positive. Chromatography and commercial DNA probes relate this species to *M. simiae* and *M. malmoense*, so 16S rRNA sequencing is actually needed for its identification [3, 15, 24]. We had good results using the GenoType HAIN Lifescience probes that do not discriminate it from *M. triplex* although the latter grows on solid media.

*In vitro* susceptibility testing is not standardized due to its difficult growth, the need of an acid pH, and its requirement of a longer time of incubation than other mycobacteria [17]. We could do it in 5 cases. All our strains, in concordance to the scientific literature [17], were resistant to isoniazid, susceptible to rifampicin and streptomycin, and with variable susceptibility to ethambutol.

In general, according to our experience and other authors', differences between infection by *M. genavense* and MAC are as follows. The first is more frequently associated with abdominal pain, with more AFB found in faeces; growth in solid medium is poor in *M. genavense* and good in MAC; *M. genavense* needs a more acid pH to grow (optimal pH 5.5), as well as a longer incubation period and addition of supplements; MAC is more resistant to standard anti-TB drugs [17].

No optimal treatment protocols for *M. genavense* disease have been reported [37], but animal models found an important reduction in AFB after 15–30 days of treatment with clarithromycin and rifabutin, after 30 days of amikacin and ethambutol, and no effect with ciprofloxacin [50]. Antimycobacterial therapy should be similar to that used in MAC infection, during several months, and must be prolonged until one year of negative culture. A combination of antibiotics should be used, among them clarithromycin, although some studies discuss the *in vitro* susceptibility of this drug [51], rifampicin, ethambutol, amikacin, moxifloxacin, and others [17]. In addition to antibiotics, immunosuppressants should be minimized. Standardized antituberculous treatment is not useful in these cases. During treatment and also similar to MAC infection, patients may develop immune reconstitution syndrome (IRIS), requiring gamma interferon [23, 52].

Outcome is poor, mainly because most patients have an underlying deep immunodeficiency. Nevertheless, survival has improved over the years—in the pre-HAART era it was around 10% in AIDS patients, while as in 2011, data of 53% survival rate at 5 years follow-up was recorded [23]. Four of our patients had good evolution, including the child who required retreatment for partial lack of compliment. The other three died during the treatment—case 1, after a month secondary to terminal AIDS; case 5, with good initial evolution, but final multiorganic failure after 8 months of treatment; and case 6, after 3 months of therapy, by pulmonary complication with respiratory insufficiency. Due to the complexity of the underlying diseases of the patients, it is difficult to determine whether any of the deaths was attributable to *M. genavense*.

In the review of non-HIV patients published from 1997 until now, we have found 29 cases of infection with *M. genavense*, mainly collected in recent years (Table 3). The prototype patient is one who, due to an underlying disease, either innate or acquired, or secondary to immunosuppressive treatments, has reached a chronic immunosuppressant situation. All were adults except a child. Nearly half of the cases [13/29] were transplant recipients and the remaining had either hematological or immunological disorders, connective diseases, or others. Most patients had disseminated infection with serious mortality [8/21], focusing on the group of transplant recipients [6/10].

In summary, *M. genavense* is an emerging opportunistic pathogen with increasingly frequency in varied immunocompromised patients. The disease mimics that of other disseminated NTM, being very similar to MAC infection, but with more abdominal involvement. There is therefore a need to obtain biopsies of enlarged lymph nodes and do a systematic analysis of stool samples, to search for AFB or nucleic acid of *M. genavense,* for early diagnosis and treatment of infection. The slow and special culture requirements are useful to confirm the diagnosis. Susceptibility testing is difficult and not always accomplished. However, treatment, although long and complex, can lead to recovery. Nevertheless, mortality remains serious.

## Figures and Tables

**Table 1:** Clinical characteristics, treatment, and outcome of the patients.

P	Age/sex	Underlying conditions	Signs and symptoms	Radiologic findings	Treatment	Outcome
1	38/M	Terminal HIV; CD4: 7/*μ*L	Fever, abdominal pain, diarrhea	CT: retroperitoneal adenopathies	CL + RIF + ETM	Death (1 month)

2	38/F	HIV (2 months); CD4: 9/*μ*L; Wilson's disease	Fever, productive cough; abdominal pain, lower limb xerosis	CXR: bilateral interstitial pattern, LUL condensation; AXR: right echogenic image	INH + RIF + PRZ	Recovery

3	35/M	HIV (<1 m); CD4: 9/*μ*L	Fever, cough, abdominal pain, enlarged cervical lymph nodes	CT: retroperitoneal and mesenteric adenopathies; mesenteric edema	AZ + ETM + AK	Recovery

4	66/M	Kidney Tx (2004); CD4: 85/*μ*L	Fever, productive cough; abdominal pain, anemia, bilateral tibiomaleolar oedema	CXR: blunting of right costophrenic angle; ARX: left echogenic image; colonoscopy: multiple adenomatous polyps	CL + ETM + LEV	Recovery

5	28/F	AML (UCBT, 2009)	Fever, anemia, abdominal pain, diarrhea, ankle and leg oedema	CXR: RUL micronodule; CT: pericardial effusion, pleural thickening; endoscopy: duodenal villous hypertrophy	First ETM + AK + LIN; then CL + RIB + ETM	Death (6 months)

6	52/F	Heart Tx (2004)	Fever, diarrhea, abdominal pain, ankle and leg oedema; lower limb petechiae	Abdominal Echo: hepatosplenomegaly, intra- and retroperitoneal lymphadenopathy; CXR: multiple bilateral infiltrates	RIF + ETM + PRZ + LEV	Death (5 months)

7	7/F	Hiper-IgE Syndrome	Fever, diarrhea, abdominal pain	CT: RLL bronchiectasies	CL + RIF + ETM + LEV	Recovery

P: patient; M: male; F: female; Tx: transplantation; AML: acute myeloid leukaemia; CT: computed tomography scan; UCBT: umbilical cord blood transplantation; CXR: chest X-ray; AXR: abdominal X-ray; LUL: left upper lobe; RLL: right lower lobe; CL: clarithromycin; INH: isoniazid; RIF: rifampicin; RIB: rifabutin; ETM: ethambutol; PRZ: pyrazinamide; AZ: azithromycin; AK: amikacin; LEV: levofloxacin; LIN: linezolid.

**Table 2:** Year, samples and microbiological characteristics of the study.

P	Year	Samples studied	ZN	Culture media growth	Samples with growth/DNA	Identification	Susceptibility testing (INH/RIF/STR/ETM/PRZ)
1	2003	Retroperitoneal adenopathy*	+	Bactec 12B and 13A	Retroperitoneal adenopathy	CONV and MB	R/S/S/I/—
2	2007	Blood (2), BAL	−	Bactec 12B and 13A	Blood and BAL	CONV and MB	R/S/S/S/—
3	2007	Retroperitoneal biopsy*, blood, faeces*, sputum	+	Bactec 12B and 13A	Retroperitoneal biopsy, blood, faeces	CONV and MB	R/S/S/S/—
4	2008	Faeces* (3), blood (4), urine (3), BM, sputum	+	Bactec 12B	Faeces	CONV and MB	R/S/S/I/—
5	2010	Faeces* (10), BM*, blood, sputum	+	MGIT and MYCO/F	Faeces, BM, blood	CONV and MB	Not done
6	2010	Faeces* (4), blood, BM*, BAS, spleen*	+	MGIT and MYCO/F	Faeces**, blood, BM**, BAS**, Spleen**	CONV and MB	R/S/S/R/S
7	2011	Faeces* (7) blood, lung biopsy*	+	MYCO/F	Faeces**, blood	CONV and MB	Not done

P: patient; BAL: bronchoalveolar lavage; BM: bone marrow; BAS: bronchoaspirate; ZN: Ziehl-Neelsen staining method; LJ: Löwenstein-Jensen; CONV: convencional; MB: molecular biology; INH: isoniazid; RIF: rifampicin; STR: streptomycin; ETM: ethambutol; PRZ: pyrazinamide; R: resistant; S: susceptible; I: intermediate.

*Samples with acid-fast bacilli.

**Samples in which DNA was detected.

**Table 3:** Cumulative cases of *M. genavense* infection in non-HIV patients.

Patient	Age/sex	Underlying condition	Disseminated disease	Outcome	Reference/year
1	47/F	Immunological disorder	Yes	Died	[20] 1997
2	38/F	Sarcoidosis chronic lymphopenia	Iliac abscess	Recovery	[21] 2000
3	80/F	CLL	Yes	Recovery	[30] 2000
4	67/F	Renal Tx	Yes	Died	[38] 2007
5	38/M	SLE	Yes	Improve	[39] 2008
6	56/M	Steam cell Tx	Duodenitis	Recovery	[27] 2009
7	55/F	SLE	Yes	Recovery	[31] 2009
8	39/M	Myasthenia gravis thymectomy	Enteritis	Recovery	[28] 2009
9	72/M	Sarcoidosis	Yes	Died	[32] 2009, [24] 2013
10	58/M	Sarcoidosis	Yes	Died	[32] 2009
11	44/M	Renal Tx	Yes	Recovery	[18] 2011
12	41/F	Renal Tx	Yes	Recovery	[23] 2011
13	63/M	Liver Tx	Yes	Died	[23] 2011
14	37/M	Heart Tx	Yes	Recovery	[40] 2008, [23] 2011
15	64/M	Renal Tx	Yes	Recovery	[29] 2011
16	43/M	Innate IL-12 deficiency	Yes	Improve*	[41] 2012, [24] 2013
17	43/F	Lung Tx	Yes	Died	[42] 2012
18	55/M	Possible RA	Pulmonar	Recovery	[24] 2013
19	57/M	Sarcoidosis	Pulmonar	Improve	[24] 2013
20	63/M	NHL	Yes	Improve*	[24] 2013
21	73/F	Renal Tx	Yes	Died	[24] 2013
22	54/F	Liver Tx	Yes	Improve*	[24] 2013
23	57/M	Interstitial nephritis	Yes	Died	[24] 2013
24	42/M	Idiopathic CD4+ lymphocytopenia	Yes	Improve*	[24] 2013
25	35/F	Autoimmune hepatitis IL-12 deficiency	Yes	Recovery	[43] 2013
26	66/M	Renal Tx	Yes	Recovery	Present study
27	28/F	AML umbilical cord Tx	Yes	Died	Present study
28	52/F	Heart Tx	Yes	Died	Present study
29	7/F	Hiper-IgE syndrome	Enteritis	Recovery	Present study

Tx: transplantation; CLL: chronic lymphocytic leukaemia; SLE: systemic lupus erythematosus; AML: acute mieloide leukaemia; RA: rheumatoid arthritis; NHL: non-Hodgkin lymphoma. *Chronic treatment.
